# Correlation of SARS-CoV-2 Viral Neutralizing Antibody Titers with Anti-Spike Antibodies and ACE-2 Inhibition among Vaccinated Individuals

**DOI:** 10.1128/spectrum.01315-22

**Published:** 2022-09-19

**Authors:** Brian Grunau, Martin Prusinkiewicz, Michael Asamoah-Boaheng, Liam Golding, Pascal M. Lavoie, Martin Petric, Paul N. Levett, Scott Haig, Vilte Barakauskas, Mohammad Ehsanul Karim, Agatha N. Jassem, Steven J. Drews, Sadaf Sediqi, David M. Goldfarb

**Affiliations:** a Centre for Health Evaluation & Outcome Sciences, St. Paul’s Hospital, Vancouver, British Columbia, Canada; b Department of Emergency Medicine, University of British Columbia, Vancouver, British Columbia, Canada; c British Columbia Emergency Health Services, Vancouver, British Columbia, Canada; d Department of Pediatrics, University of British Columbia, Vancouver, British Columbia, Canada; e Faculty of Medicine, Memorial University of Newfoundland, St. John’s, Newfoundland, Canada; f Department of Obstetrics and Gynecology, University of British Columbia, Vancouver, British Columbia, Canada; g Department of Pathology and Laboratory Medicine, University of British Columbia, Vancouver, British Columbia, Canada; h Public Health Laboratory, British Columbia Centre for Disease Control, Vancouver, British Columbia, Canada; i Canadian Blood Services, Ottawa, Ontario, Canada; j Laboratory Medicine and Pathology, University of Alberta, Edmonton, Alberta, Canada; University of Georgia

**Keywords:** neutralizing antibodies, SARS-CoV-2, COVID-19, anti-spike, ACE-2

## Abstract

SARS-CoV-2 anti-spike antibody concentrations and angiotensin converting enzyme-2 (ACE-2) inhibition have been used as surrogates to live viral neutralizing antibody titers; however, validity among vaccinated individuals is unclear. We tested the correlation of these measures among vaccinated participants, and examined subgroups based on duration since vaccination and vaccine dosing intervals. We analyzed 120 samples from two-dose mRNA vaccinees without previous COVID-19. We calculated Spearman correlation coefficients between wild-type viral neutralizing antibody titers and: anti-spike (total and IgG), anti-receptor-binding-domain (RBD), and anti-N-terminal-domain (NTD) antibodies; and ACE-2 binding by RBD. We performed three secondary analyses, dichotomizing samples by the first vaccination-to-blood collection interval, second vaccination-to-blood collection interval, and by the vaccine dosing interval (all groups divided by the median), and compared correlation coefficients (Fisher’s Z test). Of 120 participants, 63 (53%) were women, 91 (76%) and 29 (24%) received BNT162b2 and mRNA-1273 vaccines, respectively. Overall, live viral neutralization was correlated with anti-spike total antibody (correlation coefficient = 0.80), anti-spike IgG (0.63), anti-RBD IgG (0.62), anti-NTD IgG (0.64), and RBD ACE2 binding (0.65). Samples with long (>158 days) first vaccination-to-blood collection and long (>71 days) second vaccination-to-blood collection intervals demonstrated higher correlation coefficients, compared with short groups. When comparing cases divided by short (≤39 days) versus long vaccine dosing intervals, only correlation with RBD-ACE-2 binding inhibition was higher in the long group. Among COVID-negative mRNA vaccinees, anti-spike antibody and ACE-2 inhibition concentrations are correlated with live viral neutralizing antibody titers. Correlation was stronger among samples collected at later durations from vaccination.

**IMPORTANCE** Live viral neutralizing antibody titers are an accepted measure of immunity; however, testing procedures are labor-intensive. COVID-19 antibody and angiotensin converting enzyme-2 (ACE-2) levels have been used as surrogates to live viral neutralizing antibody titers; however, validity among vaccinated individuals is unclear. Using samples from 120 two-dose mRNA vaccinees without previous COVID-19, we found that live viral neutralization was correlated with COVID-19 antibody and ACE2 binding levels. When grouping samples by the time interval between vaccination and sample blood collection, samples collected over 158 days after the first vaccine and over 71 days from the second vaccine demonstrated stronger correlation between live viral neutralization titers and both antibody and ACE2 levels, in comparison to those collected earlier.

## INTRODUCTION

Severe acute respiratory syndrome-related coronavirus-2 (SARS-CoV-2), the virus that causes COVID-19, was classified as a pandemic by the World Health Organization on March 11, 2020 (1), and as of February 25, 2022 has resulted in over 5.9 million deaths (2). Substantial efforts have been undertaken to identify optimal immunization strategies to mitigate COVID-19 morbidity and mortality. Due to the resource-intensive and time-consuming nature of performing clinical trials examining outcomes of SARS-CoV-2 infections, much research has relied on surrogate immunogenicity outcomes. Detection of neutralizing antibodies to SARS-CoV-2 has been shown to correlate inversely with susceptibility to infection and COVID-19 severity, and is typically accepted as a measure of immunity (3–7).

Live viral neutralization testing is labor-intensive, requiring advanced containment, and is thus difficult for high-throughput, large volume testing. Hence, alternate strategies have been used, including measuring SARS-CoV-2 antibody concentrations of spike-related viral proteins and inhibition of viral binding onto host angiotensin converting enzyme-2 angiotensin converting enzyme-2 (ACE-2) receptors (7). However, the correlation of these measures to live viral neutralization among samples from vaccinated individuals has not been clearly established (8). Furthermore, it is unclear whether correlation between viral neutralization and other measures of immunity change with time from first vaccination, or among participants with differing vaccine dosing intervals.

For these reasons, we sought to investigate whether live viral neutralizing antibody titers correlated with both SARS-CoV-2 anti-spike protein antibody concentrations or receptor-binding domain (RBD)-ACE-2 binding inhibition. We further sought to determine if any correlation was affected by the first vaccination-to-blood collection interval, the second vaccine-to-blood collection interval, or the interval between vaccines.

## RESULTS

The full cohort included samples from 120 participants; the median age was 38 years (interquartile range [IQR] 33, 48) and 63 participants (53%) were women; 91 participants (76%) received BNT162b2 and 29 participants (34%) received mRNA-1273 (Table 1). The median first and second vaccine-to-blood collection interval was 158 days (IQR 89, 179) and 72 days (IQR 55, 131), respectively, and the median vaccine dosing interval was 39 days (IQR 25, 89). Serological outcomes did not all satisfy the D'Agostino & Pearson test for normality and, thus, correlations were calculated using the Spearman’s rank order correlation.

**TABLE 1:** Participant characteristics, overall and of subgroups classified by (i) short versus long vaccination-to-blood collection intervals, and (ii) short versus long vaccine dosing intervals

Participant characteristics	Full cohort	First vaccination-to-BC interval subgroups^*b*^		Vaccine dosing interval subgroups	
	(*n* = 120)	Short V1^*d*^-BC^*e*^ (*n* = 60)	Long V1-BC (*n* = 60)	Short VDI^*c*^ (*n* = 60)	Long VDI (*n* = 60)
Age (yr), median (IQR)	38 (33, 48)	37 (33, 47)	41 (35, 48)	38 (34, 48)	39 (33, 49)
Female sex, *n*^*g*^ (%)	63 (53)	27 (45)	36 (60)	31 (52)	32 (53)
Vaccination					
BNT162b2, *n* (%)	91 (76)	39 (65)	52 (87)	47 (78)	44 (73)
mRNA-1273, *n* (%)	29 (24)	21 (35)	8 (13)	13 (12)	16 (17)
Jan. 1/21-to-1st vaccine interval (d), median (IQR^*f*^)	12 (7, 17)	10 (7, 14)	14 (7, 19)	8 (6, 16)	13 (9, 1)
VDI interval (d^*h*^), median (IQR)	39 (25, 89)	35 (21, 42)	69 (33, 111)	25 (21, 32)	76 (42, 111)
1st vaccine-to-BC interval (d), median (IQR)	158 (89, 179)	89 (68, 109)	179 (176, 184)	150 (80, 179)	158 (99, 179)
2nd vaccine-to-BC interval (d), median (IQR)	72 (55, 131	56 (37, 73)	112 (71, 128)	123 (56, 148)	67 (53, 76)
Past medical history^*a*^					
Hypertension, *n* (%)	9 (7.5)	7 (12)	2 (3.3)	5 (8.3)	4 (6.7)
Diabetes, *n* (%)	1 (0.83)	1 (1.7)	0	1 (1.7)	0
Asthma, *n* (%)	13 (11)	7 (12)	6 (10)	8	5 (8.3)
Lung disease, *n* (%)	0	0	0	0	0
Heart disease, *n* (%)	0	0	0	0	0
Kidney disease, *n* (%)	0	0	0	0	0
Liver disease, *n* (%)	2 (1.6)	2 (3.3)	0	1 (1.7)	1 (1.7)
Cancer, *n* (%)	2 (1.6)	2 (3.3)	0	2 (3.3)	0
Hematologic disease, *n* (%)	0	0	0	0	0
Neurological disease, *n* (%)	0	0	0	0	0

a Participants answered the question “Have you been diagnosed by a physician with any of the following chronic medical conditions? (Select all that apply)”.

b First vaccination-to-BC interval, the interval (in days) between the first mRNA vaccine and the blood collection date.

c VDI, the interval (in days) between the first and second mRNA vaccine dates.

d V1, first vaccine date.

e BC, blood collection date.

f IQR, interquartile range.

g *n*, number.

h d, day.

**FIG 1:**
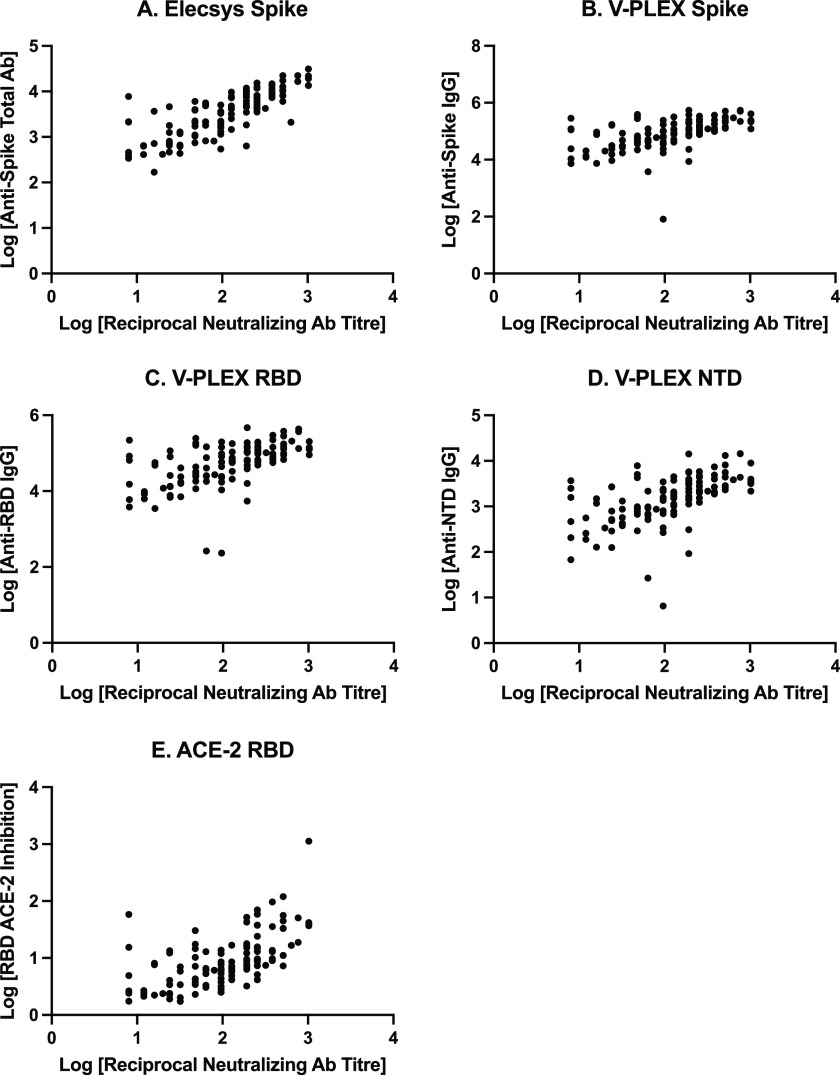
Scatterplot of the full study cohort, demonstrating relationships between live viral neutralizing antibody titers and immunogenicity measures. (A) Anti-spike total antibody concentrations (U/mL), measured on the Elecsys assay. (B) Anti-spike IgG antibody concentrations (AU/mL), measured on the V-PLEX assay. (C) Anti-receptor-binding domain (RBD) IgG antibody concentrations (AU/mL). (D) Anti-N-terminal domain (NTD) IgG antibody concentrations (AU/mL). (E) Inhibition of ACE-2 binding to RBD protein concentrations (U/mL). Ab, antibody.

Fig. 1 shows a scatterplot demonstrating the relationship between live viral neutralizing antibody titers and secondary immunogenicity outcomes. Live viral neutralizing antibody titers had a significant positive correlation (*P* < 0.0001) with all immunogenicity measures: anti-spike total antibody (Spearman’s correlation coefficient, 0.80, 95% CI = 0.72 to 0.86; anti-spike IgG, 0.63, 95% CI = 0.50 to 0.73; anti-RBD IgG, 0.62, 95% CI = 0.49 to 0.72; anti-N-terminal-domain (NTD) IgG, 0.64, 95% CI = 0.51 to 0.73; and inhibition of RBD-ACE-2 binding, 0.65, 95% CI = 0.53 to 0.75).

**FIG 2:**
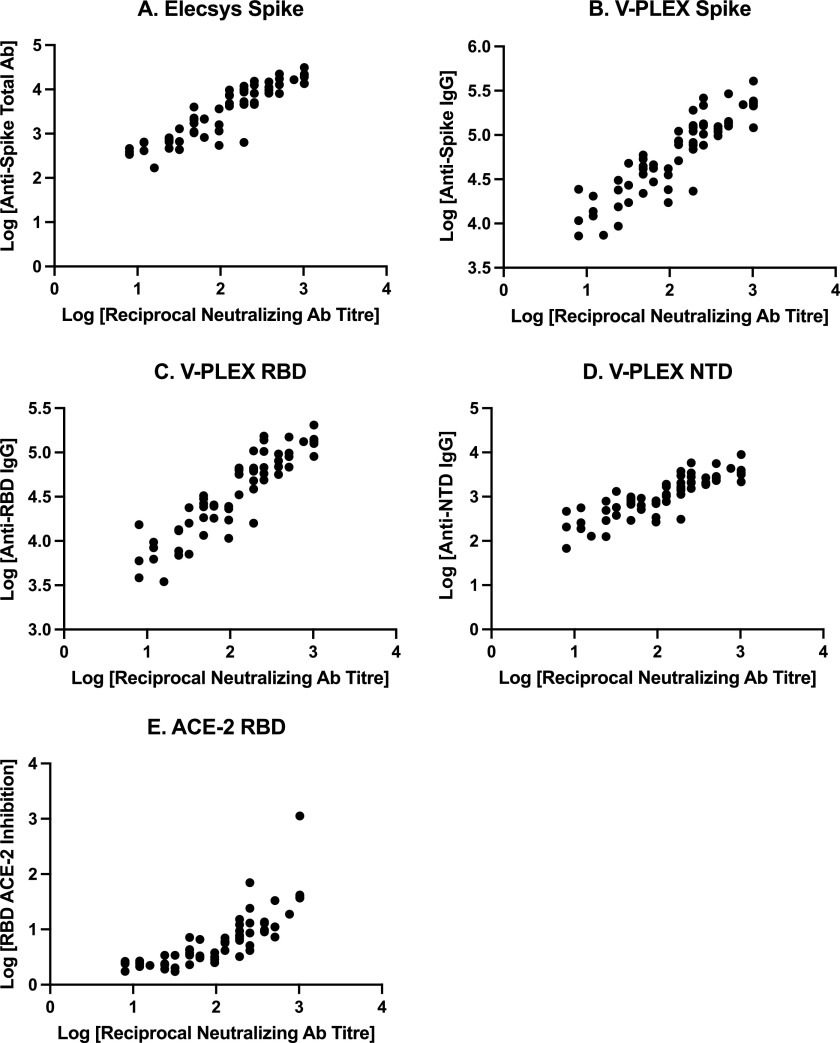
Scatterplot of the “long first vaccination-to-blood collection interval” subgroup, demonstrating relationships between live viral neutralizing antibody titers and immunogenicity measures. (A) Anti-spike total antibody concentrations (U/mL), measured on the Elecsys assay. (B) Anti-spike IgG antibody concentrations (AU/mL), measured on the V-PLEX assay. (C) Anti-receptor-binding domain (RBD) IgG antibody concentrations (AU/mL). (D) Anti-N-terminal domain (NTD) IgG antibody concentrations (AU/mL). (E) Inhibition of ACE-2 binding to RBD protein concentrations (U/mL). Ab, antibody.

Characteristics of participants in subgroups categorized by first vaccination-to-blood collection intervals as “short” (≤158 days) and “long” (>158 days) are shown in Table 1, and correlation analyses are reported in Table 2, and illustrated in Fig. S1 (“short” group) and Fig. 2 (“long” group). The median first vaccination-to-blood collection intervals in the “short” and “long” groups were 89 days (IQR 68, 109) and 179 days (IQR 176, 184), respectively. Correlation between live viral neutralizing antibody titers and other immunogenicity measures ranged from 0.49 to 0.61 in the “short” group and 0.89 to 0.91 in the “long” group (Table 2). There were significant between-group differences (*P* < 0.0001) between correlation coefficients for each immunogenicity measure.

**TABLE 2:** Correlation coefficients between viral neutralization and immunogenicity levels in subgroups based on vaccine 1-to-blood collection interval, and between-cohort comparisons

Immunogenicity measure^*a*^	Correlation coefficient (95% CI) short V1^*b*^-to-BC^*c*^ interval	Correlation coefficient (95% CI) long V1-to-BC interval	Fisher’s *Z*	*P* value
Anti-spike total	0.61 (0.42 to 0.75)	0.91 (0.85 to 0.95)	−4.37	<0.0001
Anti-spike IgG	0.49 (0.26 to 0.66)	0.89 (0.82 to 0.93)	−4.73	<0.0001
Anti-RBD IgG	0.53 (0.31 to 0.70)	0.91 (0.85 to 0.95)	−5.00	<0.0001
Anti-NTD IgG	0.51 (0.29 to 0.68)	0.88 (0.80 to 0.93)	−4.34	<0.0001
ACE-2 RBD	0.49 (0.27 to 0.67)	0.89 (0.81 to 0.93)	−4.73	<0.0001

a All measured as concentrations.

b V1, first vaccine date.

c BC, blood collection date.

Characteristics of participants in subgroups categorized by vaccine dosing intervals as “short” (≤39 days) and “long” (>39 days) are shown in Table 1, and correlation analyses are reported in Table 3 and illustrated in Fig. S2 (“short” VDI) and Fig. S3 (“long” VDI). The median vaccine dosing intervals in the “short” and “long” groups were 25 days (IQR 21, 32) and 76 days (IQR 42, 111), respectively. Correlation between live viral neutralizing antibody titers and other immunogenicity measures ranged from 0.34 to 0.50 in the “short” group and 0.50 to 0.73 in the “long” group (Table 3). There was a significant between-group difference between correlation coefficients for inhibition of RBD-ACE2 binding (*P* = 0.0058); however, a significant difference was not detected for other immunogenicity measures.

**TABLE 3:** Correlation coefficients between viral neutralization and immunogenicity levels in subgroups based on vaccine dosing interval (VDI) and between-cohort comparisons

Immunogenicity measure^*a*^	Correlation coefficient (95% CI) short VDI	Correlation coefficient (95% CI) long VDI	Fisher’s *Z*	*P* value
Anti-spike total	0.50 (0.28 to 0.67)	0.70 (0.54 to 0.82)	−1.70	0.090
Anti-spike IgG	0.37 (0.12 to 0.58)	0.63 (0.45 to 0.77)	−1.88	0.060
Anti-RBD IgG	0.38 (0.14 to 0.59)	0.50 (0.28 to 0.67)	−0.80	0.43
Anti-NTD IgG	0.34 (0.088 to 0.55)	0.55 (0.34 to 0.71)	−1.41	0.16
ACE-2 RBD	0.39 (0.15 to 0.59)	0.73 (0.58 to 0.83)	−2.76	0.0058

a All measured as concentrations.

Correlation analyses for subgroups categorized by the second vaccine-to-blood collection interval as “short” (≤71 days) and “long” (>71 days) are shown are reported in Table 4 and illustrated in Fig. S4 (“short” group) and Fig. S5 (“long” group). The median second vaccination-to-blood collection intervals in the “short” and “long” groups were 55 days (IQR 37, 64) and 123 days (IQR 82, 149), respectively. Correlation between live viral neutralizing antibody titers and other immunogenicity measures ranged from 0.43 to 0.71 in the “short” group and 0.78 to 0.86 in the “long” group (Table 4). There were significant between-group differences between correlation coefficients for each immunogenicity measure.

**TABLE 4:** Correlation coefficients between viral neutralization and immunogenicity levels in subgroups based on vaccine 2-to-blood collection interval and between-cohort comparisons

Immunogenicity measure^*a*^	Correlation coefficient (95% CI) short V2^*b*^-BC^*c*^ interval	Correlation coefficient (95% CI) long V2-BC interval	Fisher’s *Z*	*P* value
Anti-spike total	0.71 (0.56 to 0.82)	0.86 (0.77 to 0.91)	2.17	0.030
Anti-spike IgG	0.44 (0.20 to 0.63)	0.81 (0.69 to 0.88)	3.50	0.0005
Anti-RBD IgG	0.45 (0.22 to 0.64)	0.80 (0.68 to 0.88)	3.28	0.0010
Anti-NTD IgG	0.50 (0.27 to 0.67)	0.78 (0.65 to 0.87)	2.65	0.0081
ACE-2 RBD	0.43 (0.19 to 0.62)	0.79 (0.67 to 0.87)	3.26	0.0011

a All measured as concentrations.

b V2, second vaccine date.

c BC, blood collection date.

## DISCUSSION

We tested samples from participants who had received two doses of mRNA vaccine with multiple measures of immunogenicity. Overall, we found that viral neutralization was moderately positively correlated with SARS-CoV-2 antibody and RBD-ACE2 binding inhibition. Further, we found that the correlation was significantly stronger among samples collected >158 and >71 days from the first and second vaccine, respectively, in comparison with earlier collections. These data may assist further investigations by demonstrating that SARS-CoV-2 anti-spike antibody concentrations and RBD-ACE2 binding inhibition among vaccinees are correlated with more labor-intensive viral neutralization testing, and that correlation appears to improve when samples are collected at later time junctures after vaccination.

Participants in our study were from a prospective observational study of paramedics in Canada, and represented a relatively homogenous group of healthy middle-aged individuals. Groups displayed similar characteristics when divided by blood collection timing or vaccine dosing intervals. When dividing cases based on both the first and second vaccine-to-blood collection intervals, we found significant and consistent differences in correlations between measures of immunogenicity. When dividing cases based on vaccine dosing intervals, we only observed a difference in correlation for RBD-ACE2 binding inhibition. However, the “long” group tended to have higher values for each measured immunological parameter, suggesting that the vaccine dosing interval may also play a role in this relationship.

We hypothesize that antibody maturation, which may continue for multiple months after vaccination, may explain why various quantitative antibody measures demonstrated stronger correlations with live viral neutralization when examined later after vaccination, in comparison with earlier time points. This same mechanism may occur with prolonged vaccine dosing intervals, in which the second vaccine is administered to an individual with increased antibody maturity. It was recently demonstrated that antibody avidity improves considerably between the period shortly after infection to 7 to 8 months postinfection, and that avidity correlated with neutralization capacity only at 7 to 8 months after infection (9). Although we did not measure antibody avidity in our study, it is likely that a similar phenomenon occurs postvaccination with a waning of total antibody concentrations over time (10) and a concurrent antibody maturation, resulting in a stronger correlation between antibody concentration and neutralization. Given this antibody maturation, it would also suggest that measurement of spike antibody concentrations after 5 to 6 months may provide a relatively reliable surrogate marker of neutralization capacity.

There are few data among vaccinees examining the correlation between neutralizing antibody titers and other measures of immunogenicity. Using samples from 18 BNT162b2 vaccinees, Manenti et al. demonstrated a linear relationship between log-transformed viral neutralizing antibody titers and anti-spike antibody levels (8). Maeda et al. reported a Spearman’s correlation coefficient of 0.71 between neutralizing titers and S1-binding-IgG levels in samples obtained from 225 healthy two-dose BNT162b2 vaccinees on day 28 postfirst vaccine (11). Interestingly, they found that correlation declined (0.56) when examined on day 60 post-first vaccine.

Using samples from unvaccinated participants with preceding COVID-19, several studies have evaluated the correlation between neutralizing antibody titers and other measures of immunity (12). Criscuolo et al. found poor correlation between neutralizing antibody titers and antibody concentrations among 46 individuals (13), which contrasts with work from Dolscheid-Pommerich et al. which reported neutralizing antibody titers to be correlated with spike IgG concentrations (Spearman *r* = 0.82) (14), and from Tea et al. who reported that this correlation was maintained for 7 months post-COVID (15). Tan et al. and Abe et al. showed high correlation between viral neutralization and RBD-ACE2 binding inhibition among samples from participants with COVID-19 (16, 17).

Our study has several limitations. We evaluated all immune measures against a single SARS-CoV-2 strain (wild-type); however, it is possible that results may differ for other strains, particularly for those with “escape” predilection (e.g., beta or omicron). This was an observational study, and confounders may have affected our results. Differences in correlation between the two cohorts may have been affected by measured or unmeasured factors other than collection timing and vaccine dosing intervals. Our low sample size affected the precision of our results. We relied on participant self-reporting of vaccine status and dates, of which there may have been errors. We did not examine cell-mediated immunity.

In conclusion, among samples from recipients of two mRNA vaccines, SARS-CoV-2 antibody concentrations and RBD-ACE2 binding inhibition are correlated with live viral neutralization testing. Correlation was stronger among samples collected over 158 and 71 days from the first and second vaccines, respectively. Vaccine dosing intervals may also play a role in correlation between immunogenicity measures.

## MATERIALS AND METHODS

### Parent trial, study design, and participants.

Samples analyzed in this study were from the COVID-19 Occupational Risks, Seroprevalence and Immunity Among Paramedics in Canada (CORSIP) study (collected January 25, 2021 to July 14, 2021), approved by the University of British Columbia (H20-03620) and University of Toronto (40435) research ethics boards. The CORSIP study is a prospective observational cohort of adult paramedics in Canada, who provided blood samples at enrollment and 6 months (+/–10 days) after their first SARS-CoV-2 vaccine. Participants provided informed written consent, and completed sociodemographic and health questionnaires, including vaccination status and PCR/rapid antigen testing history.

This study was a *post hoc* analysis of samples that were tested with multiple serological assays and for live virus neutralizing antibody titers (18). Samples were eligible if the participant had never received a diagnosis of COVID-19 and had two doses of the same mRNA vaccine (either BNT162b2 or mRNA-1273), and the sample was nonreactive on an Elecsys Anti-SARS-CoV-2 nucleocapsid (Roche, IN, USA) assay (19) to confirm absence of prior SARS-CoV-2 infection.

### Outcome measures.

All outcome measures were log transformed due to the skewed nature of immunogenicity data (20, 21). The primary outcome measure was the reciprocal of live virus neutralizing antibody titers measured against the wild-type SARS-COV-2/Canada/VIDO-01/2020 strain (22). Secondary outcomes included anti-spike wild-type strain total antibody concentrations, measured with the Elecsys Anti-SARS-Cov-2 S assay (Roche, IN, USA) (23); IgG antibody concentrations against spike, RBD, and NTD wild-type antigens, measured with the V-PLEX COVID-19 Coronavirus Panel 2 IgG assay (Meso Scale Discovery [MSD], MD, USA); and inhibition of viral RBD binding onto host ACE-2 receptor for the wild-type strain, measured with the V-PLEX SARS-COV2 Panel 11 ACE-2 kit (MSD, MD, USA). See supplemental materials for further details on testing procedures.

### Statistical analysis.

Statistical analyses were performed using Prism GraphPad version 9.2.0 (CA, USA) and R (Foundation for Statistical Computing, Vienna, version 3.2.4). We reported participant characteristics as median (with IQR) for continuous variables, and counts (with percentage) for categorical variables. Outcome measures were log-transformed for analyses. We planned to assess Pearson correlation coefficients (with 95% CI) between the primary outcome and each secondary outcome if all demonstrated a normal distribution (based on the D'Agostino & Pearson test), or otherwise calculate Spearman rank correlation coefficients (with 95% CI).

We performed three secondary analyses. First, as the interval between vaccination and sample collection may affect correlation between immunogenicity measures, we compared correlation coefficients from subgroups classified as “short” versus “long” first vaccination-to-blood collection intervals. We divided groups based on the median value in the cohort, and calculated correlation coefficients between live viral neutralization antibody titers and each secondary outcome. We then compared correlation coefficients from the two subgroups to determine if there were significant differences, using a 2-tailed Fisher’s Z test in the Concor package for R (24). Second, as vaccine dosing intervals have been shown to impact immune response (10) (and thus may modify correlation between immunogenicity measures), we similarly divided the full cohort into two groups, classified as “short” versus “long” vaccine dosing interval (divided by the median value of the overall cohort) groups, and compared correlation coefficients. Third, we repeated the analysis after dividing the full cohort by the median second vaccination-to-blood collection interval, categorizing as “short” versus “long.”

## References

[1] Tedros A. 2020. WHO Director-General’s opening remarks at the media briefing on COVID-19–11 March 2020. https://www.who.int/director-general/speeches/detail/who-director-general-s-opening-remarks-at-the-media-briefing-on-covid-19---11-march-2020. Accessed 19 June 2021.

[2] World Health Organization. 2021. WHO coronavirus (COVID-19) dashboard. https://covid19.who.int Accessed 25 February 2022.

[3] McMahan K, Yu J, Mercado NB, Loos C, Tostanoski LH, Chandrashekar A, Liu J, Peter L, Atyeo C, Zhu A, Bondzie EA, Dagotto G, Gebre MS, Jacob-Dolan C, Li Z, Nampanya F, Patel S, Pessaint L, Van Ry A, Blade K, Yalley-Ogunro J, Cabus M, Brown R, Cook A, Teow E, Andersen H, Lewis MG, Lauffenburger DA, Alter G, Barouch DH. 2021. Correlates of protection against SARS-CoV-2 in rhesus macaques. Nature 590:630–634. doi:10.1038/s41586-020-03041-6. 33276369 PMC7906955

[4] Dispinseri S, Secchi M, Pirillo MF, Tolazzi M, Borghi M, Brigatti C, De Angelis ML, Baratella M, Bazzigaluppi E, Venturi G, Sironi F, Canitano A, Marzinotto I, Tresoldi C, Ciceri F, Piemonti L, Negri D, Cara A, Lampasona V, Scarlatti G. 2021. Neutralizing antibody responses to SARS-CoV-2 in symptomatic COVID-19 is persistent and critical for survival. Nat Commun 12:2670. doi:10.1038/s41467-021-22958-8. 33976165 PMC8113594

[5] Feng S, Phillips DJ, White T, Sayal H, Aley PK, Bibi S, Dold C, Fuskova M, Gilbert SC, Hirsch I, Humphries HE, Jepson B, Kelly EJ, Plested E, Shoemaker K, Thomas KM, Vekemans J, Villafana TL, Lambe T, Pollard AJ, Voysey M, Oxford COVID Vaccine Trial Group. 2021. Correlates of protection against symptomatic and asymptomatic SARS-CoV-2 infection. Nat Med 27:2032–2040. doi:10.1038/s41591-021-01540-1. 34588689 PMC8604724

[6] Bergwerk M, Gonen T, Lustig Y, Amit S, Lipsitch M, Cohen C, Mandelboim M, Levin EG, Rubin C, Indenbaum V, Tal I, Zavitan M, Zuckerman N, Bar-Chaim A, Kreiss Y, Regev-Yochay G. 2021. Covid-19 breakthrough infections in vaccinated health care workers. N Engl J Med 385:1474–1484. doi:10.1056/NEJMoa2109072. 34320281 PMC8362591

[7] Zhu F, Althaus T, Tan CW, Costantini A, Chia WN, Van Vinh Chau N, Van Tan L, Mattiuzzo G, Rose NJ, Voiglio E, Wang L-F. 2022. WHO international standard for SARS-CoV-2 antibodies to determine markers of protection. Lancet Microbe 3:e81–e82. doi:10.1016/S2666-5247(21)00307-4. 34901897 PMC8641955

[8] Manenti A, Gianchecchi E, Dapporto F, Leonardi M, Cantaloni P, Fattorini F, Piu P, Bollati V, Pastorino U, Apolone G, Sozzi G, Montomoli E. 2022. Evaluation and correlation between SARS-CoV-2 neutralizing and binding antibodies in convalescent and vaccinated subjects. J Immunol Methods 500:113197. doi:10.1016/j.jim.2021.113197. 34843712 PMC8619878

[9] Pichler D, Baumgartner M, Kimpel J, Rössler A, Riepler L, Bates K, Fleischer V, von Laer D, Borena W, Würzner R. 2021. Marked increase in avidity of SARS-CoV-2 antibodies 7–8 months after infection is not diminished in old age. J Infect Dis 224:764–770. doi:10.1093/infdis/jiab300. 34086960 PMC8195195

[10] Grunau B, Asamoah-Boaheng M, Lavoie PM, Karim ME, Kirkham TL, Demers PA, Barakauskas V, Marquez AC, Jassem AN, O’Brien SF, Drews SJ, Haig S, Cheskes S, Goldfarb DM. 2021. A higher antibody response is generated with a 6- to 7-week (vs standard) severe acute respiratory syndrome coronavirus 2 (SARS-CoV-2) vaccine dosing interval. Clin Infect Dis 75:e888–e891. doi:10.1093/cid/ciab938. PMC8690265 34849655

[11] Maeda K, Amano M, Uemura Y, Tsuchiya K, Matsushima T, Noda K, Shimizu Y, Fujiwara A, Takamatsu Y, Ichikawa Y, Nishimura H, Kinoshita M, Matsumoto S, Gatanaga H, Yoshimura K, Oka S, Mikami A, Sugiura W, Sato T, Yoshida T, Shimada S, Mitsuya H. 2021. Correlates of neutralizing/SARS-CoV-2-S1-binding antibody response with adverse effects and immune kinetics in BNT162b2-vaccinated individuals. Sci Rep 11:22848. doi:10.1038/s41598-021-01930-y. 34819514 PMC8613264

[12] Lamikanra A, Nguyen D, Simmonds P, Williams S, Bentley EM, Rowe C, Otter AD, Brooks T, Gilmour K, Mai A, Dadhra J, Csatari M, Ziyenge S, Oliveira M, Ploeg R, Tsang P, Zambon M, Gopal R, Xiao JH, Townsend A, Roberts D, Harvala H. 2021. Comparability of six different immunoassays measuring SARS-CoV-2 antibodies with neutralizing antibody levels in convalescent plasma: from utility to prediction. Transfusion 61:2837–2843. doi:10.1111/trf.16600. 34342366 PMC8447482

[13] Criscuolo E, Diotti RA, Strollo M, Rolla S, Ambrosi A, Locatelli M, Burioni R, Mancini N, Clementi M, Clementi N. 2021. Weak correlation between antibody titers and neutralizing activity in sera from SARS-CoV-2 infected subjects. J Med Virol 93:2160–2167. doi:10.1002/jmv.26605. 33064340 PMC7675753

[14] Dolscheid Pommerich R, Bartok E, Renn M, Kümmerer BM, Schulte B, Schmithausen RM, Stoffel-Wagner B, Streeck H, Saschenbrecker S, Steinhagen K, Hartmann G. 2022. Correlation between a quantitative anti-SARS-CoV-2 IgG ELISA and neutralization activity. J Med Virol 94:388–392. doi:10.1002/jmv.27287. 34415572 PMC8426838

[15] Tea F, Ospina Stella A, Aggarwal A, Ross Darley D, Pilli D, Vitale D, Merheb V, Lee FXZ, Cunningham P, Walker GJ, Fichter C, Brown DA, Rawlinson WD, Isaacs SR, Mathivanan V, Hoffmann M, Pöhlman S, Mazigi O, Christ D, Dwyer DE, Rockett RJ, Sintchenko V, Hoad VC, Irving DO, Dore GJ, Gosbell IB, Kelleher AD, Matthews GV, Brilot F, Turville SG. 2021. SARS-CoV-2 neutralizing antibodies: longevity, breadth, and evasion by emerging viral variants. PLoS Med 18:e1003656. doi:10.1371/journal.pmed.1003656. 34228725 PMC8291755

[16] Tan CW, Chia WN, Qin X, Liu P, Chen MI-C, Tiu C, Hu Z, Chen VC-W, Young BE, Sia WR, Tan Y-J, Foo R, Yi Y, Lye DC, Anderson DE, Wang L-F. 2020. A SARS-CoV-2 surrogate virus neutralization test based on antibody-mediated blockage of ACE2–spike protein–protein interaction. Nat Biotechnol 38:1073–1078. doi:10.1038/s41587-020-0631-z. 32704169

[17] Abe KT, Li Z, Samson R, Samavarchi-Tehrani P, Valcourt EJ, Wood H, Budylowski P, Dupuis AP, Girardin RC, Rathod B, Wang JH, Barrios-Rodiles M, Colwill K, McGeer AJ, Mubareka S, Gommerman JL, Durocher Y, Ostrowski M, McDonough KA, Drebot MA, Drews SJ, Rini JM, Gingras A-C. 2020. A simple protein-based surrogate neutralization assay for SARS-CoV-2. JCI Insight 5 doi:10.1172/jci.insight.142362. PMC7566699 32870820

[18] Grunau B, Goldfarb DM, Asamoah-Boaheng M, Golding L, Kirkham TL, Demers PA, Lavoie PM. 2022. Immunogenicity of extended mRNA SARS-CoV-2 vaccine dosing intervals. JAMA 327:279. doi:10.1001/jama.2021.21921. 34860253 PMC8642809

[19] Ainsworth M, Andersson M, Auckland K, Baillie JK, Barnes E, Beer S, Beveridge A, Bibi S, Blackwell L, Borak M, Bown A, Brooks T, Burgess-Brown NA, Camara S, Catton M, Chau KK, Christott T, Clutterbuck E, Coker J, Cornall RJ, Cox S, Crawford-Jones D, Crook DW, D'Arcangelo S, Dejnirattsai W, Dequaire JMM, Dimitriadis S, Dingle KE, Doherty G, Dold C, Dong T, Dunachie SJ, Ebner D, Emmenegger M, Espinosa A, Eyre DW, Fairhead R, Fassih S, Feehily C, Felle S, Fernandez-Cid A, Fernandez Mendoza M, Foord TH, Fordwoh T, Fox McKee D, Frater J, Gallardo Sanchez V, Gent N, Georgiou D, Groves CJ, et al. 2020. Performance characteristics of five immunoassays for SARS-CoV-2: a head-to-head benchmark comparison. Lancet Infect Dis 20:1390–1400. doi:10.1016/S1473-3099(20)30634-4. 32979318 PMC7511171

[20] Bošnjak B, Stein SC, Willenzon S, Cordes AK, Puppe W, Bernhardt G, Ravens I, Ritter C, Schultze-Florey CR, Gödecke N, Martens J, Kleine-Weber H, Hoffmann M, Cossmann A, Yilmaz M, Pink I, Hoeper MM, Behrens GMN, Pöhlmann S, Blasczyk R, Schulz TF, Förster R. 2021. Low serum neutralizing anti-SARS-CoV-2 S antibody levels in mildly affected COVID-19 convalescent patients revealed by two different detection methods. Cell Mol Immunol 18:936–944. doi:10.1038/s41423-020-00573-9. 33139905 PMC7604543

[21] Khoury DS, Cromer D, Reynaldi A, Schlub TE, Wheatley AK, Juno JA, Subbarao K, Kent SJ, Triccas JA, Davenport MP. 2021. Neutralizing antibody levels are highly predictive of immune protection from symptomatic SARS-CoV-2 infection. Nat Med 27:1205–1211. doi:10.1038/s41591-021-01377-8. 34002089

[22] Zakhartchouk AN, Liu Q, Petric M, Babiuk LA. 2005. Augmentation of immune responses to SARS coronavirus by a combination of DNA and whole killed virus vaccines. Vaccine 23:4385–4391. doi:10.1016/j.vaccine.2005.04.011. 16005746 PMC7115501

[23] Cobas. 2021. Elecsys Anti-SARS-CoV-2 S V 1.0. https://www.rochecanada.com/content/dam/rochexx/roche-ca/products/docs/package_inserts/Anti-SARS-CoV-2-S-09289267190-EN-Can.pdf. Accessed 10 August 2021.

[24] Diedenhofen B, Musch J. 2015. cocor: a comprehensive solution for the statistical comparison of correlations. PLoS One 10:e0121945. doi:10.1371/journal.pone.0121945. 25835001 PMC4383486

