# Supplementary material for: Correlation of SARS-CoV-2 Viral Neutralizing Antibody Titers with Anti-Spike Antibodies and ACE-2 Inhibition among Vaccinated Individuals
Source: Microbiol Spectr. 2022 Sep 19;10(5):e01315-22. doi: 10.1128/spectrum.01315-22 (PMC9602390; doi:10.1128/spectrum.01315-22)
Supplement: Supplemental file 1 — Download spectrum.01315-22-s0001.pdf, PDF file, 0.5 MB [file spectrum.01315-22-s0001.pdf]

SUPPLEMENTAL MATERIALS

Neutralization Antibody Testing Procedures

Live virus neutralizing antibody titers were measured against the Wuhan "wild type" SARS-COV-2/Canada/VIDO-01/2020 strain. This standard virus neutralization assay, as previously described,[1] was performed at the Biosafety Level 3 laboratory at the British Columbia Centre for Disease Control (Petric and Levett). Each serum specimen was heat inactivated at 56 °C for 30 minutes and duplicate serial 2-fold dilutions from 1:8-1:4096 were each incubated with 100 TCID50 of SARS-CoV-2 for 2 hours, then added-monolayers of Vero-E6 cells. Monolayers were examined after 72 hours for characteristic cytopathogenic effect (CPE). The inverse of the highest serum dilution-inhibit CPE was deemed the antibody titre. As each sample was tested twice, the mean value was used in the analysis.

V-PLEX RBD-ACE2 Binding Inhibition Testing Procedures

Inhibition of human ACE-2 receptor binding onto viral spike and RBD was measured using the V- PLEX SARS-COV2 Panel 11 ACE-2 kits (Meso Scale Discovery [MSD], MD, USA; reported as Units per mL), for the Wuhan virus strain. The MSD assay was performed as per the manufacturer's instructions, at the BC Children's Hospital Research Institute (Golding, Prusinkiewicz, and Lavoie). Samples were tested at a 1:20 dilution, with the diluent provided by the manufacturer (Diluent 100). For samples with results above the detection limit, re-testing was performed at a 1:40 dilution.

V-PLEX Spike and Receptor-Binding Domain Antibody Testing Procedures

Anti-S1 spike, Anti-Receptor Binding Domain (RBD), and Anti-N-terminal domain (NTD) IgG antibody concentrations were measured using the V-PLEX COVID-19 Coronavirus Panel 2 IgG assay (MSD, MD, USA; reported as Arbitrary Units per mL]). The MSD assay was performed as per the manufacturer's instructions, at the BC Children's Hospital Research Institute (Golding and Lavoie). Samples were tested at a 1:10,000 dilution, with the diluent provided by the manufacturer (Diluent 100). Heat inactivation was not performed. The assay includes a standard curve based on a reference standard that contains a pre-determined concentration of each antigen. It also includes three serological controls which contain a known concentration of IgG antibodies against the antigens of the assay. We ran the standard curve and serological controls on every plate-assess for quality control.

Elecsys Spike and Nucleocapsid Antibody Testing Procedures

Elecsys assays were performed at the Canadian Blood Services national COVID-19 research laboratory (Drews and O'Brien). Anti-S1 spike total antibody concentrations were measured with the quantitative Elecsys Anti-SARS-Cov-2 S assay (Roche, IND, USA).[2] The assay was performed as per manufacturer's instructions, with lot-to-lot standardization and assay calibration as per Roche standard operating procedures and manufacturer's instructions.

Samples with an anti-S concentration above the measuring range (250 U/mL) were diluted by the Roche analyzer with Universal Diluent at 1:400. After dilution by the analyzer, the software automatically utilized the dilution value when calculating the sample concentration. Heat inactivation was not performed as was not part of the manufacturer's standard protocol. For quality control, PreciControl Anti-SARS-CoV-2 was used as per manufacturer's instructions. These were run at least daily prior-any testing (e.g., every 24 hours when test is in use). Cut-offs

were determined automatically by the analyzer software based on calibrated master curves. For calibration, the method is standardized against an internal Roche standard for anti- SARS-CoV-2 provided with the assay (N and S). A pre-defined master curve is adapted-the analyzer using the kit calibration reagents. Calibration is performed as per manufacturer's instructions. All serum samples were also similarly tested with the Elecsys Anti-SARS-CoV-2 nucleocapsid (Roche, IND, USA) assay,[3] an immunoassay for the in-vitro qualitative detection of nucleocapsid antibodies (including IgG)-SARS-CoV-2, in order-determine eligibility.

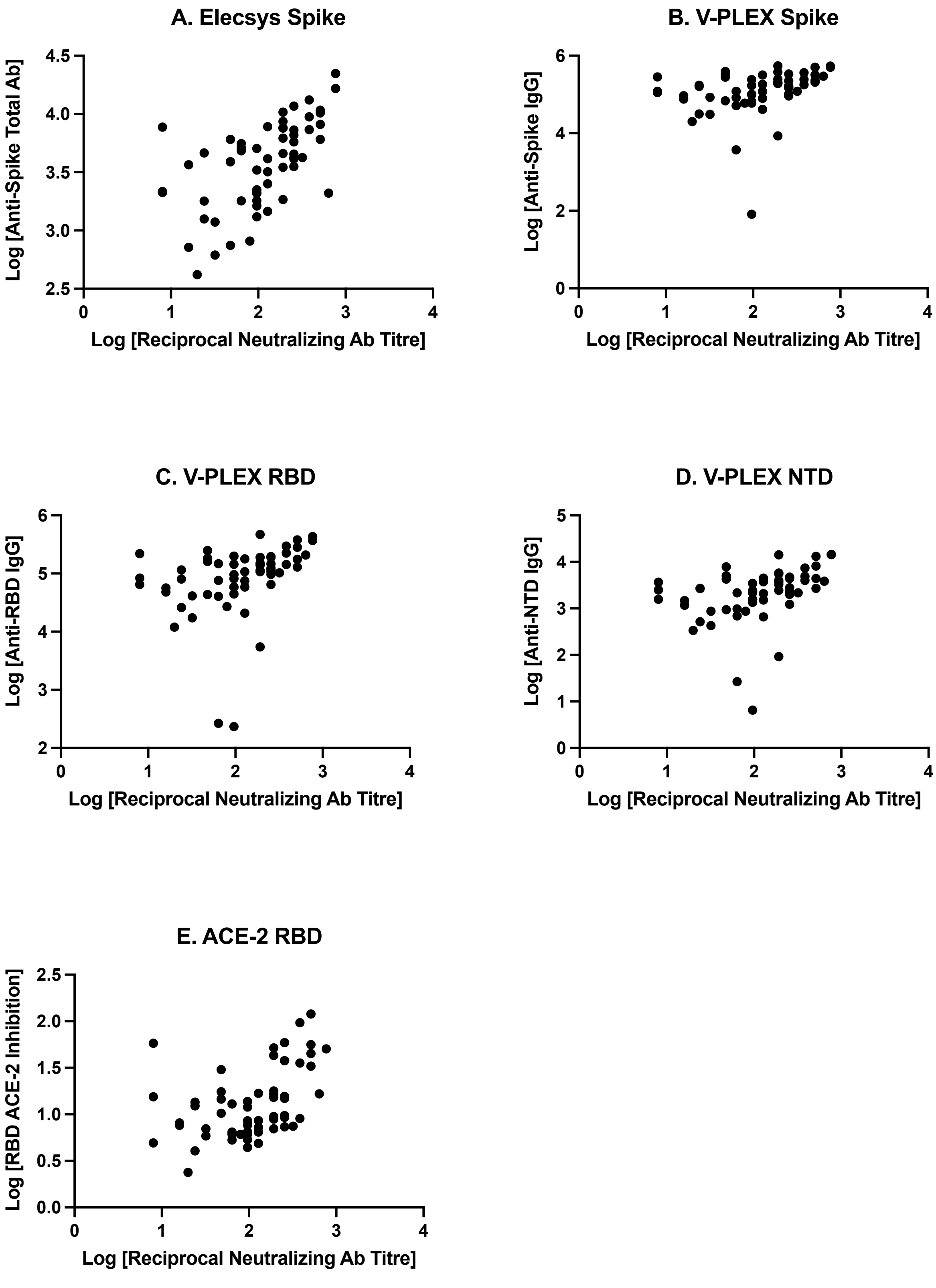

Supplemental Figure 1: Scatterplot of the “Short First Vaccination-to-Blood Collection interval” Subgroup, Demonstrating Relationships between Live Viral Neutralizing Antibody Titres and Immunogenicity Measures

A. Anti-Spike Total Antibody Concentrations (U/mL), measured on the Elecsys assay.
B. Anti-Spike IgG Antibody Concentrations (AU/mL), measured on the V-PLEX assay.
C. Anti-Receptor-Binding Domain (RBD) IgG Antibody Concentrations (AU/mL)
D. Anti-N-Terminal Domain (NTD) IgG Antibody Concentrations (AU/mL)
E. Inhibition of ACE-2 binding to RBD Protein Concentrations (U/mL)
Ab, antibody

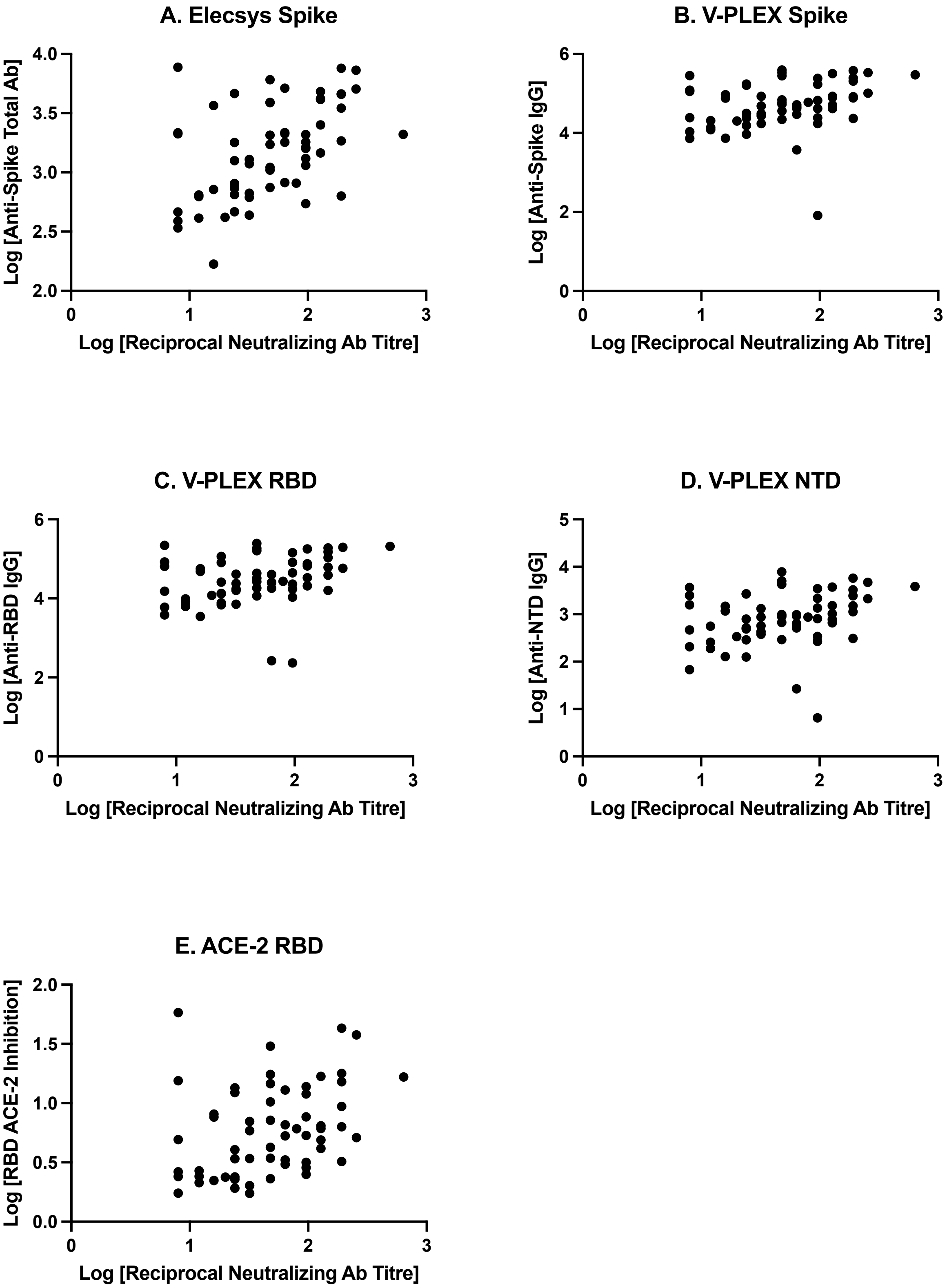

Supplemental Figure 2: Scatterplot of the "Short Vaccine Dosing interval" Subgroup, Demonstrating Relationships between Live Viral Neutralizing Antibody Titres and Immunogenicity Measures

A. Anti-Spike Total Antibody Concentrations (U/mL), measured on the Elecsys assay.
B. Anti-Spike IgG Antibody Concentrations (AU/mL), measured on the V-PLEX assay.
C. Anti-Receptor-Binding Domain (RBD) IgG Antibody Concentrations (AU/mL)
D. Anti-N-Terminal Domain (NTD) IgG Antibody Concentrations (AU/mL)
E. Inhibition of ACE-2 binding to RBD Protein Concentrations (U/mL)
Ab, antibody

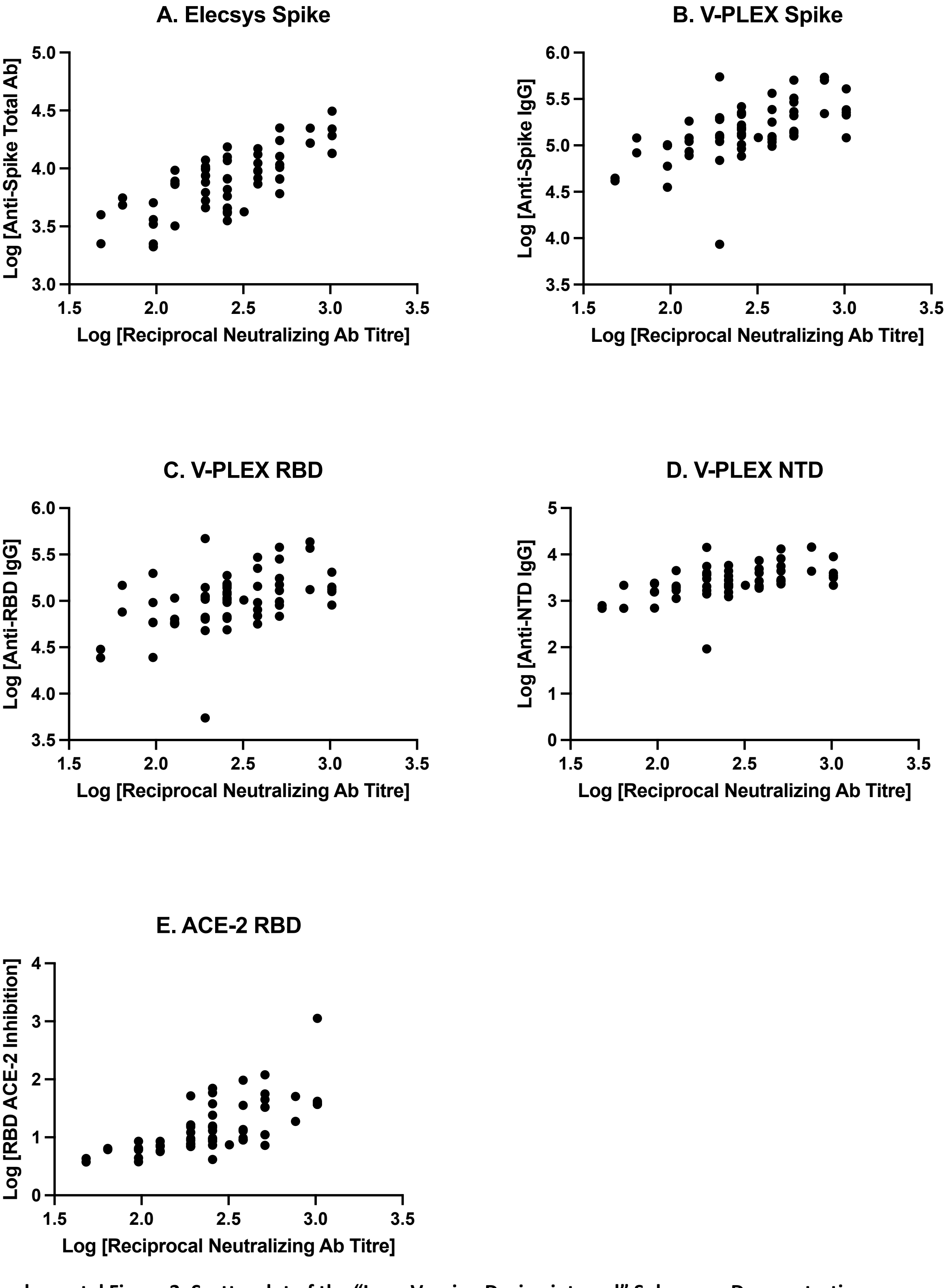

Supplemental Figure 3: Scatterplot of the "Long Vaccine Dosing interval" Subgroup, Demonstrating Relationships between Live Viral Neutralizing Antibody Titres and Immunogenicity Measures

A. Anti-Spike Total Antibody Concentrations (U/mL), measured on the Elecsys assay.

B. Anti-Spike IgG Antibody Concentrations (AU/mL), measured on the V-PLEX assay.

C. Anti-Receptor-Binding Domain (RBD) IgG Antibody Concentrations (AU/mL)

D. Anti-N-Terminal Domain (NTD) IgG Antibody Concentrations (AU/mL)

E. Inhibition of ACE-2 binding to RBD Protein Concentrations (U/mL)

Ab, antibody

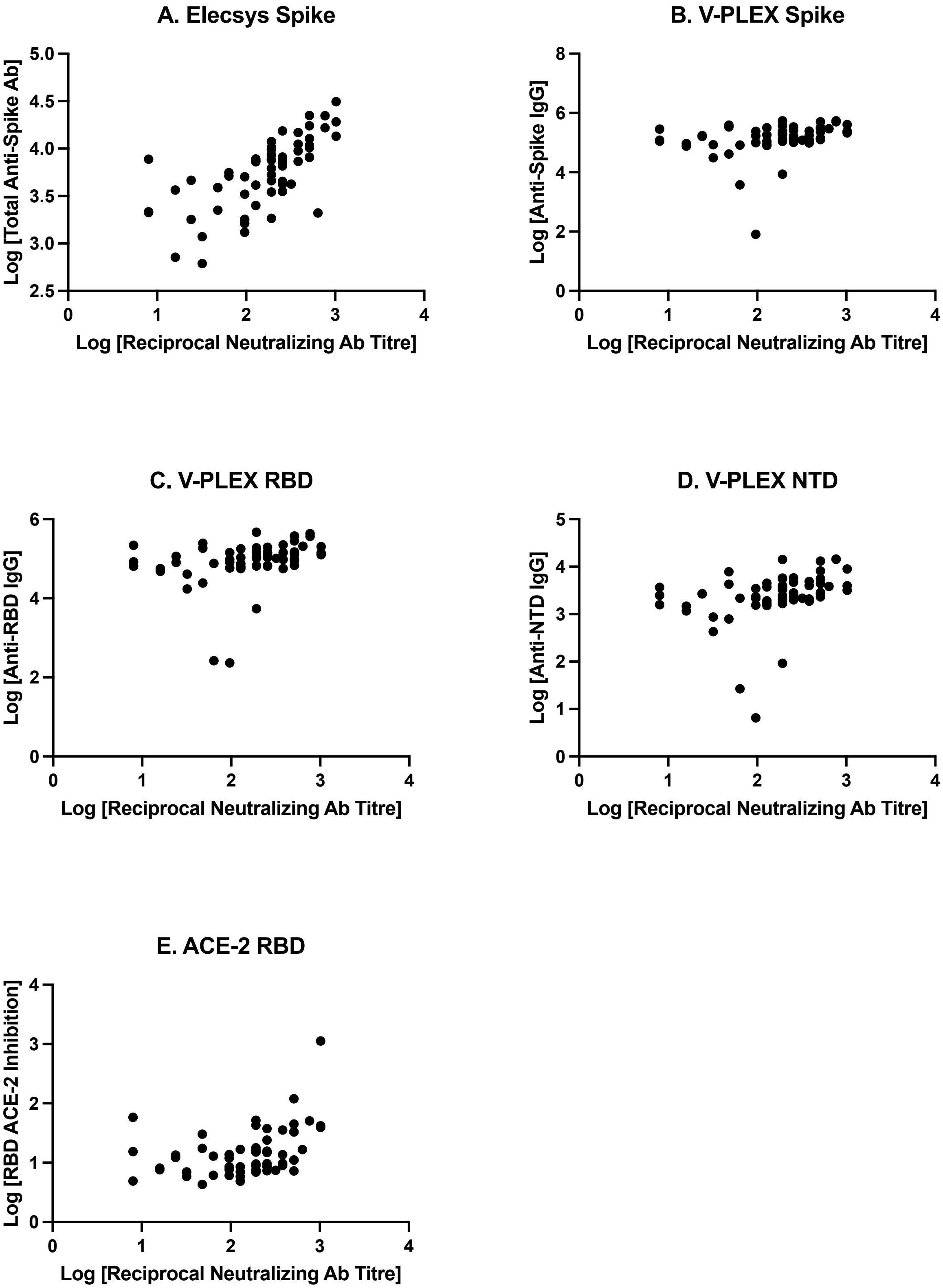

Supplemental Figure 4: Scatterplot of the "Short Second Vaccination-to-Blood Collection interval" Subgroup, Demonstrating Relationships between Live Viral Neutralizing Antibody Titres and Immunogenicity Measures

A. Anti-Spike Total Antibody Concentrations (U/mL), measured on the Elecsys assay.
B. Anti-Spike IgG Antibody Concentrations (AU/mL), measured on the V-PLEX assay.
C. Anti-Receptor-Binding Domain (RBD) IgG Antibody Concentrations (AU/mL)
D. Anti-N-Terminal Domain (NTD) IgG Antibody Concentrations (AU/mL)
E. Inhibition of ACE-2 binding to RBD Protein Concentrations (U/mL)
Ab, antibody

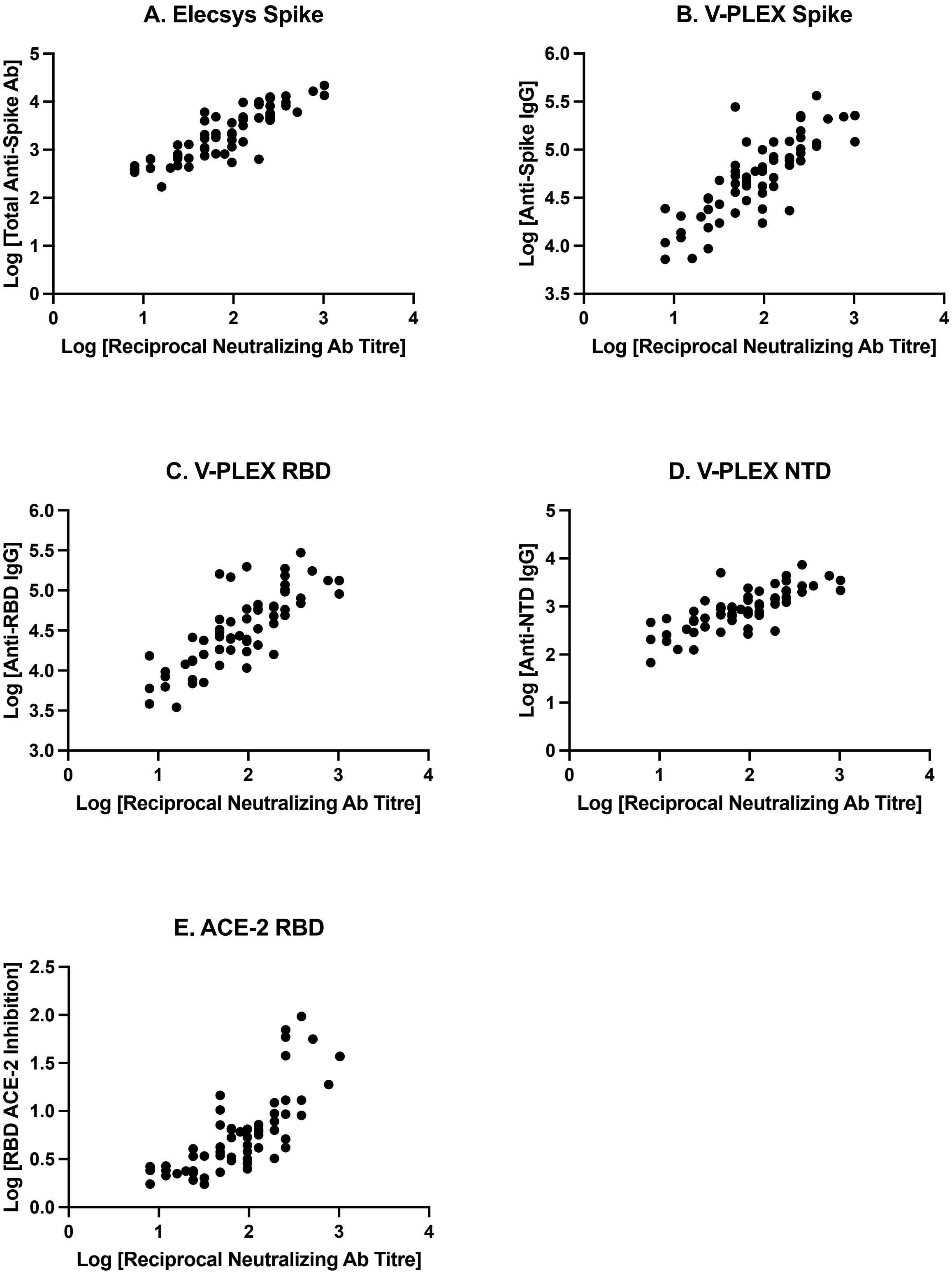

Supplemental Figure 5: Scatterplot of the "Long Second Vaccination-to-Blood Collection interval" Subgroup, Demonstrating Relationships between Live Viral Neutralizing Antibody Titres and Immunogenicity Measures

A. Anti-Spike Total Antibody Concentrations (U/mL), measured on the Elecsys assay.

B. Anti-Spike IgG Antibody Concentrations (AU/mL), measured on the V-PLEX assay.

C. Anti-Receptor-Binding Domain (RBD) IgG Antibody Concentrations (AU/mL)

D. Anti-N-Terminal Domain (NTD) IgG Antibody Concentrations (AU/mL)

E. Inhibition of ACE-2 binding to RBD Protein Concentrations (U/mL)

Ab, antibody

References

1. Zakhartchouk AN, Liu Q, Petric M, Babiuk LA. Augmentation of immune responses to SARS coronavirus by a combination of DNA and whole killed virus vaccines. *Vaccine*. 2005;23(35):4385-4391. doi:10.1016/j.vaccine.2005.04.011.
2. Cobas. Elecsys Anti-SARS-CoV-2 S V 1.0. 2021. https://www.rochecanada.com/content/dam/rochexx/roche-ca/products/docs/package_inserts/Anti-SARS-CoV-2-S-09289267190-EN-Can.pdf. Accessed August 10, 2021.
3. Ainsworth M, Andersson M, Auckland K, et al. Performance characteristics of five immunoassays for SARS-CoV-2: a head-to-head benchmark comparison. *Lancet Infect Dis*. 2020;20(12):1390-1400. doi:10.1016/S1473-3099(20)30634-4.
